# PARP-1 depletion in combination with carbon ion exposure significantly reduces MMPs activity and overall increases TIMPs expression in cultured HeLa cells

**DOI:** 10.1186/s13014-016-0703-x

**Published:** 2016-09-22

**Authors:** Atanu Ghorai, Asitikantha Sarma, Priyanka Chowdhury, Utpal Ghosh

**Affiliations:** 1 Department of Biochemistry & Biophysics, University of Kalyani, Kalyani, 741235 India; 2 Inter-University Accelerator Center (IUAC), Aruna Asaf Ali Marg, New Delhi, 110067 India; 3 Present address: Department of Biological Sciences, Tata Institute of Fundamental Research (TIFR), Homi Bhabha Road, Colaba, Mumbai, 400005 India

**Keywords:** Carbon ion exposure, Gamma radiation, PARP-1, Matrix metalloproteinases (MMPs), Tissue inhibitor of matrix metalloproteinases (TIMPs), Cell death, Apoptosis

## Abstract

**Background:**

Hadron therapy is an innovative technique where cancer cells are precisely killed leaving surrounding healthy cells least affected by high linear energy transfer (LET) radiation like carbon ion beam. Anti-metastatic effect of carbon ion exposure attracts investigators into the field of hadron biology, although details remain poor. Poly(ADP-ribose) polymerase-1 (PARP-1) inhibitors are well-known radiosensitizer and several PARP-1 inhibitors are in clinical trial. Our previous studies showed that PARP-1 depletion makes the cells more radiosensitive towards carbon ion than gamma. The purpose of the present study was to investigate combining effects of PARP-1 inhibition with carbon ion exposure to control metastatic properties in HeLa cells.

**Methods:**

Activities of matrix metalloproteinases-2, 9 (MMP-2, MMP-9) were measured using the gelatin zymography after 85 MeV carbon ion exposure or gamma irradiation (0- 4 Gy) to compare metastatic potential between PARP-1 knock down (HsiI) and control cells (H-vector - HeLa transfected with vector without shRNA construct). Expression of MMP-2, MMP-9, tissue inhibitor of MMPs such as TIMP-1, TIMP-2 and TIMP-3 were checked by immunofluorescence and western blot. Cell death by trypan blue, apoptosis and autophagy induction were studied after carbon ion exposure in each cell-type. The data was analyzed using one way ANOVA and 2-tailed paired-samples T-test.

**Results:**

PARP-1 silencing significantly reduced MMP-2 and MMP-9 activities and carbon ion exposure further diminished their activities to less than 3 % of control H-vector. On the contrary, gamma radiation enhanced both MMP-2 and MMP-9 activities in H-vector but not in HsiI cells. The expression of MMP-2 and MMP-9 in H-vector and HsiI showed different pattern after carbon ion exposure. All three TIMPs were increased in HsiI, whereas only TIMP-1 was up-regulated in H-vector after irradiation. Notably, the expressions of all TIMPs were significantly higher in HsiI than H-vector at 4 Gy. Apoptosis was the predominant mode of cell death and no autophagic death was observed.

**Conclusions:**

Our study demonstrates for the first time that PARP-1 inhibition in combination with carbon ion synergistically decreases MMPs activity along with overall increase of TIMPs. These data open up the possibilities of improvement of carbon ion therapy with PARP-1 inhibition to control highly metastatic cancers.

**Electronic supplementary material:**

The online version of this article (doi:10.1186/s13014-016-0703-x) contains supplementary material, which is available to authorized users.

## Background

Metastasis is the advance stage of cancer progression with high morbidity leading to death of patients. In metastatic stage, cancer cells spread from their primary sites to the neighboring cells or to a distant organ by achieving migratory and invasiveness properties. Finally, they become able to get direct access to blood and lymphatic vessels [1–3]. During this tumor invasion and metastasis, several matrix metalloproteinases (MMPs) like MMP-2 and MMP-9 play a critical role by degrading the extracellular matrix and components of the basement membrane [4–6]. These two are gelatinase (which can degrade gelatin) in nature and are found in large quantities in various cancer tissues suggesting their role in cancer progression [7–10]. Activity of MMPs is one of the major determining factors of metastasis. Regulation of their activities is precisely maintained at the level of transcription, activation of pro-MMP precursor zymogens and inhibition by endogenous inhibitors, called tissue inhibitors of metalloproteinases (TIMPs) [11, 12]. Literature review expresses that overexpression and hyperactivity of MMP-2 and MMP-9 has been observed in pre-cancer and cancer lesions of all kinds of cancers including the uterine cervical cancer which is second most common malignant tumor among female in the developing countries [13–22]. Detailed studies are needed to elucidate the molecular mechanism of cancer progression and thereby search for the better molecular targets to control such aggressive form of cancer.

Radiotherapy mainly by low linear energy transfer (LET) radiation like X-rays & gamma rays have been an important treatment tool for many malignant tumors but it has a number of limitations. One of the limitations is - such type of ionizing radiation can enhance the metastatic nature in various kinds of cancer cells by increasing the expression as well as activities of MMP-2 and MMP-9 leading to great difficulties in radiotherapy. A number of studies showed the increased expression and activities of MMP-2 and MMP-9 after X-ray irradiation [23–25]. Up-regulation of MMP-2 and MMP-9 activity was also observed in TGF-beta 1 transgenic mouse model after thoracic irradiation [26]. Protein expression level of TIMP-1 and TIMP-2 was also found to be altered in a complex manner after gamma or X-rays implicating that MMPs and TIMPs are of great concern in radiotherapy [27–29]. High LET particle radiations are very much useful and advantageous over low LET radiations for radiotherapy purposes due to its characteristic energy deposition at Bragg’s Peak and dose-depth distribution, higher relative biological effectiveness (RBE) value, independent of oxygen requirement and cell cycle phases etc [30]. Such charged particles produce huge double strand DNA breaks in a close proximity - known as ‘cluster DNA damage’ unlike to low LET radiation and consequently have different downstream DNA damage responses from low LET radiation [31]. High LET charged particles not only show higher cell killing but also inhibit MMPs activities and help to block cancer metastasis although detailed mechanism is still not clear [32–34].

One of the immediate responses after DNA damage by ionizing radiation is the activation of poly(ADP-ribosyl)ation reaction predominantly by the poly(ADP-ribose) polymerase-1 or PARP-1, a well- known DNA base excision repair protein [35]. It has diversified roles in various biological and physiological processes like- maintenance of chromatin structure, cell cycle regulation, transcription regulation, apoptosis, inflammation etc [36]. Since PARP-1 is involved in DNA repair process, its inhibitors have been used as chemo-sensitizer or radio-sensitizer and are in clinical trial to treat BRCA1/2 mutant breast cancer cells [37, 38]. There are various PARP-1 inhibitors that are in clinical trial for different kinds of cancers [39–42]. The role of PARP-1 in ‘cluster DNA damage’ condition by high LET radiation is still under investigation. Inhibition of PARP-1 shows higher radio-sensitization towards high LET carbon ion beam than low LET gamma radiation as reported by us as well as Takahisa et al. [43, 44]. Furthermore, we showed that knocking down of PARP-1 gene by shRNA can trigger several apoptotic parameters leading to more apoptosis after ‘cluster DNA damage’ by high LET carbon beam [45]. There is a substantial number of studies indicating a cross talk between PARP-1 and matrix metalloproteinases (MMPs). Inhibition of PARP by pharmacological inhibitor or siRNA can down-regulate MMP-9 and MMP-2 expressions as well as their enzymatic activities [46–48]. But cross talk between PARP-1 and MMPs/TIMPs in cells irradiated with carbon ion beam is not known. Here, we have investigated the status of MMPs and TIMPs in cultured HeLa cells irradiated with carbon ion beam in combination with PARP-1 depletion.

## Materials and methods

### Chemical and antibodies

Bovine serum albumin (BSA), trypan blue, ethidium bromide, paraformaldehyde were purchased from Sigma-Aldrich, USA; Sodium dodecyl sulfate (SDS) from Boeringer Mannheim, Germany; Trypsin and hygromycin B from HiMedia, India; Triton X-100 and glycine from SRL, India. Other bio-chemicals and reagents of molecular biology grade used in this study were purchased locally.

The primary antibodies were purchased from Santa Cruz Biotechnology Inc, USA such as mouse anti-PARP-1 [sc-74469], mouse anti-MMP-2 [sc-80201], mouse anti-MMP-9 [sc-58389], mouse anti-TIMP-1 [sc-80365], mouse anti-TIMP-2 [sc-80366] and mouse anti-TIMP-3 [sc-80367]. Rabbit anti-beta actin [ab151526] was from Abcam, UK. Mouse anti-MMP-2 antibody [Cat No- 436000] and rabbit anti-caspase-3 [Cat No- 700182] were purchased from Invitrogen, USA. Secondary antibodies used for immunofluorescence (IF) were goat anti-mouse IgG_1_-FITC [sc-2078], donkey anti-mouse IgG-FITC [sc-2099] and donkey anti-rabbit IgG-R [sc-2095] from Santa Cruz Biotechnology Inc., USA. Anti-mouse IgG, HRP-linked antibody [Product#7076] was obtained from Cell Signaling Technology, Inc., USA for western blot. VisGlow plus Chemiluminescent Substrate of HRP (Visual Protein Biotechnology Corp, Taiwan) was used for developing western blots.

### Cell culture

Human cervical cancer cell line HeLa was obtained from National Centre for Cell Sciences (NCCS), Pune, India. HeLa cells were grown in DMEM supplemented with 10 % bovine serum (complete medium) at 37 °C in humidified atmosphere containing 5 % CO_2_.

### Preparation of PARP-1 knocked down cells

PARP-1 knocked down HeLa cells were prepared using lipofectamine 2000^TM^ (Invitrogen, USA) mediated stable transfection procedure with shRNA construct against PARP-1 in plasmid pRNA-U6.1 (GeneScript, USA) followed by our earlier method [45]. PARP-1 shRNA transfected cells are denoted as HsiI and the negative control cells (only vector plasmid without shRNA insert was used for transfection) are termed as H-vector. All experiments using transfected cells were done within 3-4 passages of cells.

### Irradiation with carbon ion and gamma ray

We used the Radiation Biology Laboratory of Inter University Accelerator Centre (IUAC), New Delhi, India to grow the cells and subsequently irradiated it with carbon ion beam in the heavy ion irradiation facility ASPIRE as mentioned in [49]. Cells were seeded at a density of 0.5 × 10^6^ cells/plate on 35 mm petri dishes (Nunc, USA). The energy of carbon ion beam from the Pelletron Accelerator was 85 MeV (equivalent to 7.08 MeV/nucleon). The energy of the beam on the cell surface was 62 MeV (equivalent 5.16 MeV/nucleon) with entrance LET 287 keV/μm as calculated by TRIM software. The beam flux was maintained at about 2 × 10^5^ particles/cm^2^/s. The dose in Gy was calculated using the standard relation given below, where the cell is taken to be water equivalent.

$$ \mathrm{Dose}\ \left(\mathrm{Gy}\right) = 1.6 \times {10}^{\hbox{-} 9} \times \mathrm{LET}\ \left(\mathrm{keV}/\upmu \mathrm{m}\right) \times \mathrm{Fluence}\ \left(\mathrm{particles}/{\mathrm{cm}}^2\right). $$

The irradiation dose range used was from 0 to 4 Gy, where 0 Gy stood for the control sample. Prior to the exposure to the ion beam the cells were grown for 10–12 h inside the CO_2_ incubator till they reached 50–60 % confluent. Immediately after irradiation, cells were taken back to bio-safety cabinet and washed with phosphate buffered saline (PBS) for three times. Then 1 ml serum free medium for zymography experiment otherwise 2 ml serum free medium was added to the each plate and incubated in CO_2_ incubator for further 18–20 h. The samples were then prepared for the zymography or processed for other experiments following the methods as described later.

The cells were also irradiated with different doses (0- 4 Gy) of gamma ray [Co^60^] at Saha Institute of Nuclear Physics, Kolkata, India as mentioned in [43]. The dose rate was 0.47 Gy/min. After irradiation cells were processed according to the procedure as described above for respective experiments.

### Total cell death by trypan blue

Total cell death after carbon ion exposure (0- 4 Gy) followed by 19 h incubation in serum free medium was measured by dye exclusion method using 0.2 % trypan blue. The blue colored cells denote dead cells. Percentages of dead cells were calculated and plotted.

### Study of MMP-2 & MMP-9 activities by gelatin zymography

Gelatin zymography was done to assess MMP-2 and MMP-9 activity [50]. Almost 40 % confluent cells (for H-vector and HsiI) were seeded for 10–12 h growth in complete medium. After irradiation with carbon ion or gamma ray (0- 4 Gy) the cells were washed thrice with PBS and incubated in 1 ml of serum free medium for 19 h. Now, these conditioned medium were collected, centrifuged to remove cellular debris, and concentrated by centrifugation through Amicon membrane (Millipore), with a cutoff of 30 kDa. Then protein concentrations were determined by Lowry’s method. Equal amount of protein was subjected to 0.1 % gelatin 8 % SDS-PAGE under non-reducing conditions. The gel was run for 2 h at 4 °C until the bromophenol blue dye comes out from the gel. Then gel was washed in 2.5 % Triton X-100 for 1 h with constant shaking and washed with double distilled water for three times with 20 min interval in shaking condition. After that gel was incubated in developing buffer (Tris-Cl_2_, pH- 7.5, NaCl, CaCl_2_ and Brij-35) for 30 min shaking at room temperature and finally the gel was kept in fresh developing buffer for overnight at 37 °C. After 23 h incubation gels were stained with 0.1 % Coomassie blue for 1 h and destained in 10 % methanol and 10 % acetic acid. Zones of gelatinolytic activity were detected as clear bands against a blue background. A parallel SDS-PAGE without gelatin was run loaded with same amount of sample under same experimental condition and was treated as internal loading control (shown in Additional file 1) with respect to which the band intensities of zymography was normalized and plotted in bar diagram.

### Protein expression of PARP-1, MMP-2, MMP-9, TIMP-1, TIMP-2 & TIMP-3 by immunofluorescence (IF) study

Protein expression of PARP-1, MMP-2, MMP-9, TIMP-1, TIMP-2 & TIMP-3 with and without carbon ion exposure (0- 4 Gy) in both H-vector and HsiI cells was studied by standard IF technique as described in our recent article [45]. Cells were processed after 19 h of irradiation. Images were captured using monochrome camera in AxioScope AI of Carl Zeiss Fluorescence Microscope. Then these images were converted into RGB mode by putting pseudo-colour using Zen software of Carl Zeiss, Germany. Intensities were measured by ImageJ software [45, 51]. Expression level of these proteins were then normalized against the expression of beta-actin. Expression of respective protein in un-irradiated control H-vector was considered as 100 % to calculate the protein expression in all the data point in a respective experiment. For each data point at least 250 cells were analyzed.

The primary antibodies used were mouse anti-PARP-1 (1:150), mouse anti-MMP-2 (1:200), mouse anti-MMP-9 (1:200), mouse anti-TIMP-1 (1:200), mouse anti-TIMP-2 (1:200), mouse anti-TIMP-3 (1:200) and rabbit anti-beta actin (1:900). Secondary antibodies used were goat anti-mouse IgG_1_-FITC, donkey anti-mouse IgG-FITC and donkey anti-rabbit IgG-R (Rhodamine tagged). The dilution for secondary antibodies was 1:400.

### Western blotting for MMP-2

Whole cell lysate was prepared for western blotting using the method as described in [52]. In brief, after 19 h post-carbon ion or gamma exposure (0- 4 Gy) cells were washed with PBS twice followed by trypsinization and again washed with 1 ml of wash buffer (HEPES-KOH 10 mM, pH 7.5, MgCl_2_ 1.5 mM, KCl 10 mM, DTT 1 mM). Then cell lysis was done using the lysis buffer (Tris-Cl 10 mM pH 7.5, MgCl_2_ 1 mM, EGTA 1 mM, β-mercaptoethanol 0.75 %, CHAPS 0.5 %, glycerol 10 %) in ice for 30 min. Clear supernatant was obtained after centrifugation at 12,000 g for 15 min at 4 °C. Protein content was estimated by standard Lowry’s method. 30 μg of protein was separated in standard 10 % SDS-PAGE in 1X Tris glycine buffer (25 mM Tris, 250 mM glycine, 0.1 % SDS) at 120 V. After completion of electrophoretic run, the protein gel was processed for immunoblotting as our earlier report with slight modification [52]. The concentrations of antibodies used were mouse anti-MMP-2 antibody (1:2500), rabbit anti-beta actin (1:10000) and HRP-conjugated secondary antibody (1:5000). Finally, the blot was developed using VisGlow plus Chemiluminescent Substrate of HRP. The signals from the blots were captured by Typhoon 9210 – Variable Mode Imager (GE Healthcare, USA).

### Autophagy detection by IF

Autophagy is a self-eating process and this kind of cell death can be triggered under several cellular stresses like starvation, pH change etc. including DNA damages. Autophagolysosomes or autolysosomes, double-membrane bound vesicles like structures, are produced in the cells to engulf the intracellular damaged organelles or aggregated proteins by lysosome-mediated process and finally degraded by lysosomal hydrolases [53, 54]. Here, LC3B protein plays a critical role for proper processing of autophagy. Generally, this protein is found in cytosol, but during autophagy this protein localizes to the autophagolysosomes or autolysosomes. Such phenomenon is used as a marker for autophagic membrane. We used LC3B Antibody Kit for Autophagy (Molecular Probes, Invitrogen, USA) detection following the protocol given by the manufacturer through IF technique [55]. Chloroquine diphosphate, an inducer of autophagy, was also used as positive control as supplied by the manufacturer.

### Apoptosis as detected by activation of caspase-3 and nuclear fragmentation

Caspase-3 activation in both H-vector and HsiI cells after carbon ion exposure (0- 4 Gy) was detected through IF following the procedure as described in our previous work [45]. Here the antibody used for caspase-3 detection was specific for the cleaved active form of caspase-3 with dilution 1:300. Cells were processed after 20 h of post-irradiation incubation. Donkey anti-rabbit IgG-R (Rhodamine) was used as secondary antibody. We also measured caspase-3 activity by fluorimetric study. After 16- 18 h growth, cells were irradiated with carbon ion exposure (0- 4 Gy) and further incubated in serum free medium for another 20 h. Cells were counted by Countess (Invitrogen, USA) after trypsinization. Two million cells were taken to assay the caspase-3 activity using the protocol provided by the manufacturer (ApoAlert® Caspase 3 Fluorescence Assay Kit from Clontech, USA) and also described in our previous study [43]. Apoptosis was also detected by visualizing fragmented nucleus or apoptotic body formation under fluorescence microscope as per our earlier method [43].

### Image analysis

All images were analyzed using ImageJ software [45, 51]. Fluorescence intensities of the cells were measured by ImageJ software and then it was calculated as intensity per cell in all experiments and plotted.

### Statistical analysis

We evaluated the statistical significance for the irradiated samples of each cell type with respect to the un-irradiated control of each cell type using one way ANOVA with Dunnett’s test from IBM SPSS Statistics Version 21 software. The significance values were denoted as ‘*’ (0.01 < *p* ≤ 0.05), ‘**’ (0.001 < *p* ≤ 0.01) and ‘***’ (*p* ≤ 0.001). We did 2-tailed paired-samples T-test using the same software to calculate the statistical significance between H-vector and HsiI cells at a particular dose and significance values were denoted as ‘#’ (0.01 < *p* ≤ 0.05), ‘##’ (0.001 < *p* ≤ 0.01) and ‘###’ (*p* ≤ 0.001) and also mentioned in the text. Each experiment was repeated at least three times unless otherwise specified.

## Results

### PARP-1 expression in H-vector and HsiI cells

**Fig. 1:**
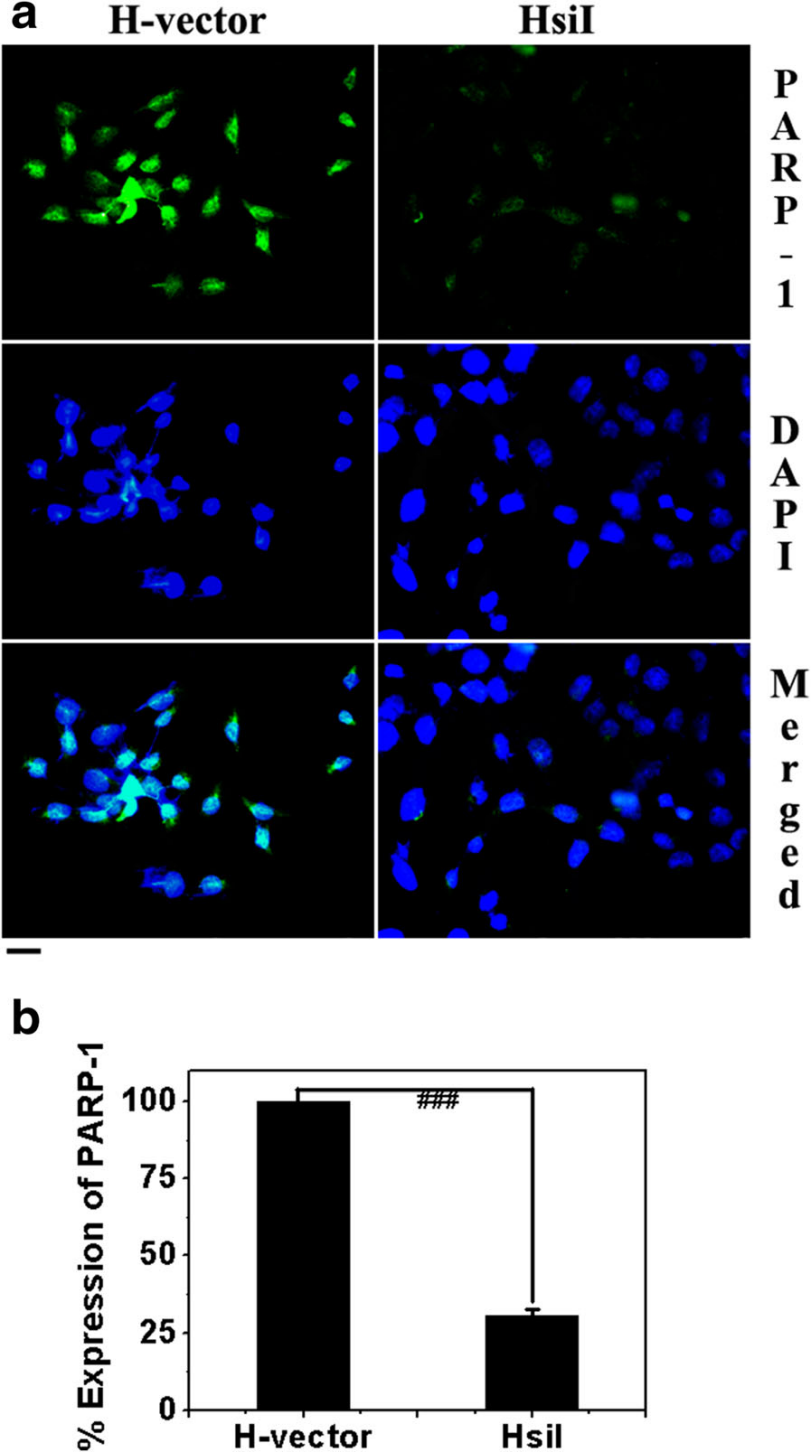
Checking of PARP-1 expression by IF study. **a** Typical photographs of immunostained H-vector & HsiI cells are given. Anti-PARP-1 antibody was detected by the secondary antibodies of FITC tagged goat anti-mouse IgG_1_. DAPI was used to counterstain the nucleus. Scale bar represents 20 μm. **b** Relative expression of PARP-1 was calculated with respect to beta actin by measuring fluorescence intensities by ImageJ software. PARP-1 expression in H-vector was plotted as 100 % protein expression and accordingly PARP-1 level in HsiI was calculated and plotted. This experiment was repeated four times. ‘###’ represents *p* ≤ 0.001

The protein expression of PARP-1 in knocked down cells (HsiI) as well as in control H-vector was checked by standard immunofluorescence (IF) technique as shown in Fig. 1a. The expression level of PARP-1 was normalized with respect to internal control beta actin. Normalized PARP-1 expression in H-vector was taken as 100 % protein expression and subsequently PARP-1 level in HsiI was calculated and plotted in Fig. 1b. Almost 75 % inhibition of PARP-1 expression was observed in HsiI cells compared to H-vector.

### Activity of MMP-2 and MMP-9 drastically reduces upon PARP-1 depletion and it further diminishes when exposed with carbon ion but not with gamma irradiation

**Fig. 2:**
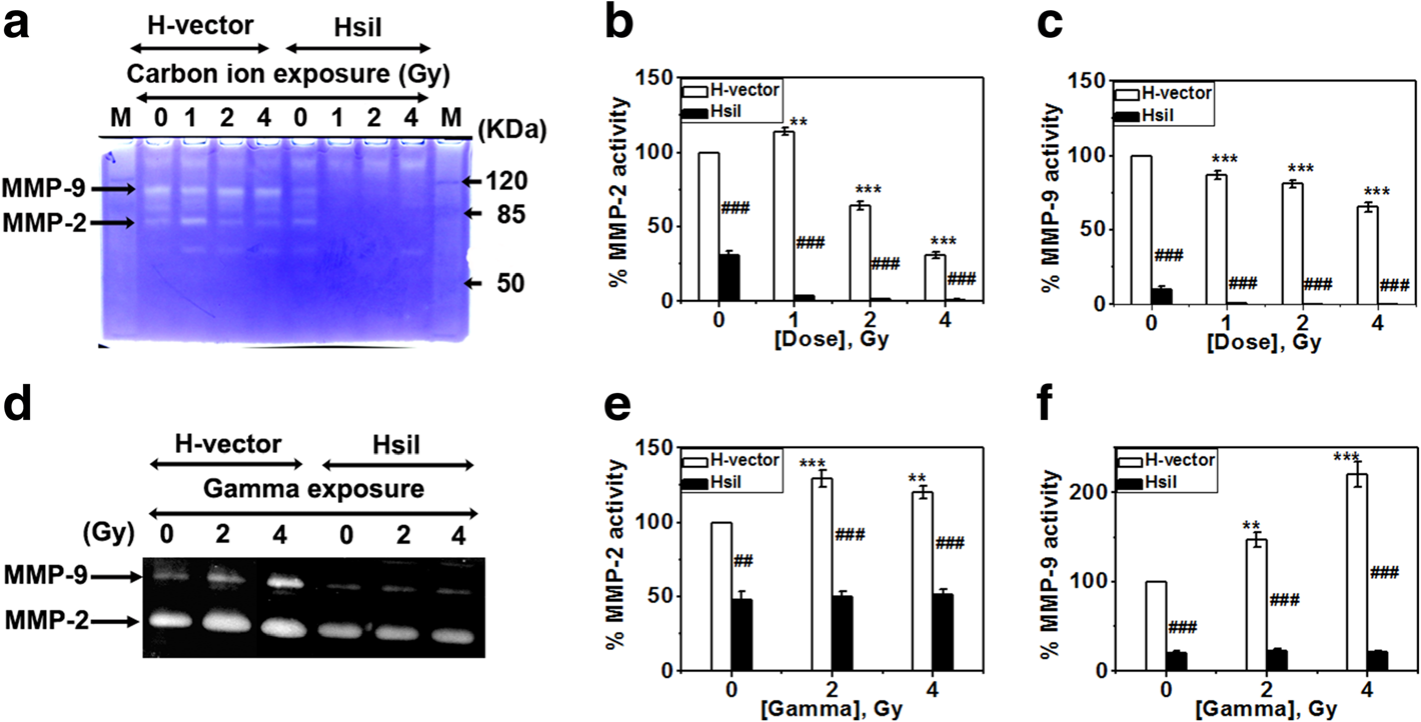
Activity of MMP-2 and MMP-9 by gelatin zymography after carbon ion exposure and gamma (0- 4 Gy) irradiation. **a** Typical photograph of gelatin zymogram after 19 h of post-carbon ion exposure. The clear bands of MMP-9 (92 KDa) and MMP-2 (72 KDa) signify their gelatinolytic activity after irradiation. ‘M’ marked lanes represent molecular weight marker. **b & c** The intensities of bands were measured by ImageJ software, normalized with loading control (given as Additional file 1) and plotted. The intensities of the representative band of both MMP-2 & MMP-9 in un-irradiated H-vector were considered as 100 % activity and accordingly the activity of the respective MMPs at different doses were calculated and plotted. **d, e & f** MMP-2 and MMP-9 activities are shown in H-vector and HsiI after gamma irradiation and representative bar diagram was prepared following the procedure as mentioned above. ‘***’ on respective bar denotes *p* ≤ 0.001 for activity at that dose compared with un-irradiated respective cell-type H-vector or HsiI. ‘###’ denotes *p* ≤ 0.001 for difference of MMPs activities between H-vector and HsiI at a particular dose

MMP-2 and MMP-9 activities were detected by gelatin zymography. A typical gelatin zymogram after carbon ion and gamma exposure (0- 4 Gy) in both H-vector and HsiI cells was shown in Fig. 2a and d respectively. Zones of gelatinolytic activity of both MMP-2 and MMP-9 were found as clear bands with blue background (Fig. 2a and d). Intensities of the bands representative to MMP-2 and MMP-9 were measured using ImageJ software. Band intensity of un-irradiated H-vector was taken as 100 % gelatinolytic activity (both MMP-2 and MMP-9) and accordingly activity at different dose of carbon ion or gamma exposure with and without depletion of PARP-1 was calculated and plotted as shown in Fig. 2b, c, e and f. All the data were normalized using loading control as shown in Additional file 1. Carbon ion exposure reduced activity of the both MMPs in a dose-dependent manner in H-vector cells as shown in Fig. 2a, although extent of reduction was higher for MMP-2. For example, MMP-2 and MMP-9 activity in H-vector cells was reduced to almost 30 % and 70 % at 4 Gy of carbon ion exposure. Notably, about 75 % depletion of PARP-1 expression reduced about 70 % activity of MMP-2 and about 90 % activity of MMP-9 in un-irradiated cells. Carbon ion exposure further drastically reduced their activity lower than 3 % in PARP-1 depleted cells as shown in Fig. 2a, b and c. So, knocking down of PARP-1 gene synergized high LET carbon ion exposure induced reduction of activities of MMP-2 and MMP-9.

On the contrary, low LET gamma radiation enhanced the activities of MMP-2 and MMP-9 in H-vector (0- 4 Gy) as shown in Fig. 2d. The %activities are shown in Fig. 2e and f. Depletion of PARP-1 reduced the MMP-2 and MMP-9 activities as usual as shown in Fig. 2d. After gamma irradiation HsiI cells did not show any significant enhancement of neither MMP-2 nor MMP-9 in our experimental condition as shown in Fig. 2e and f respectively. It implies that PARP-1 inhibition can protect the HeLa cells from being metastatic after low LET gamma irradiation.

### Role of PARP-1 on expression of MMP-2 and MMP-9 after carbon ion exposure

**Fig. 3:**
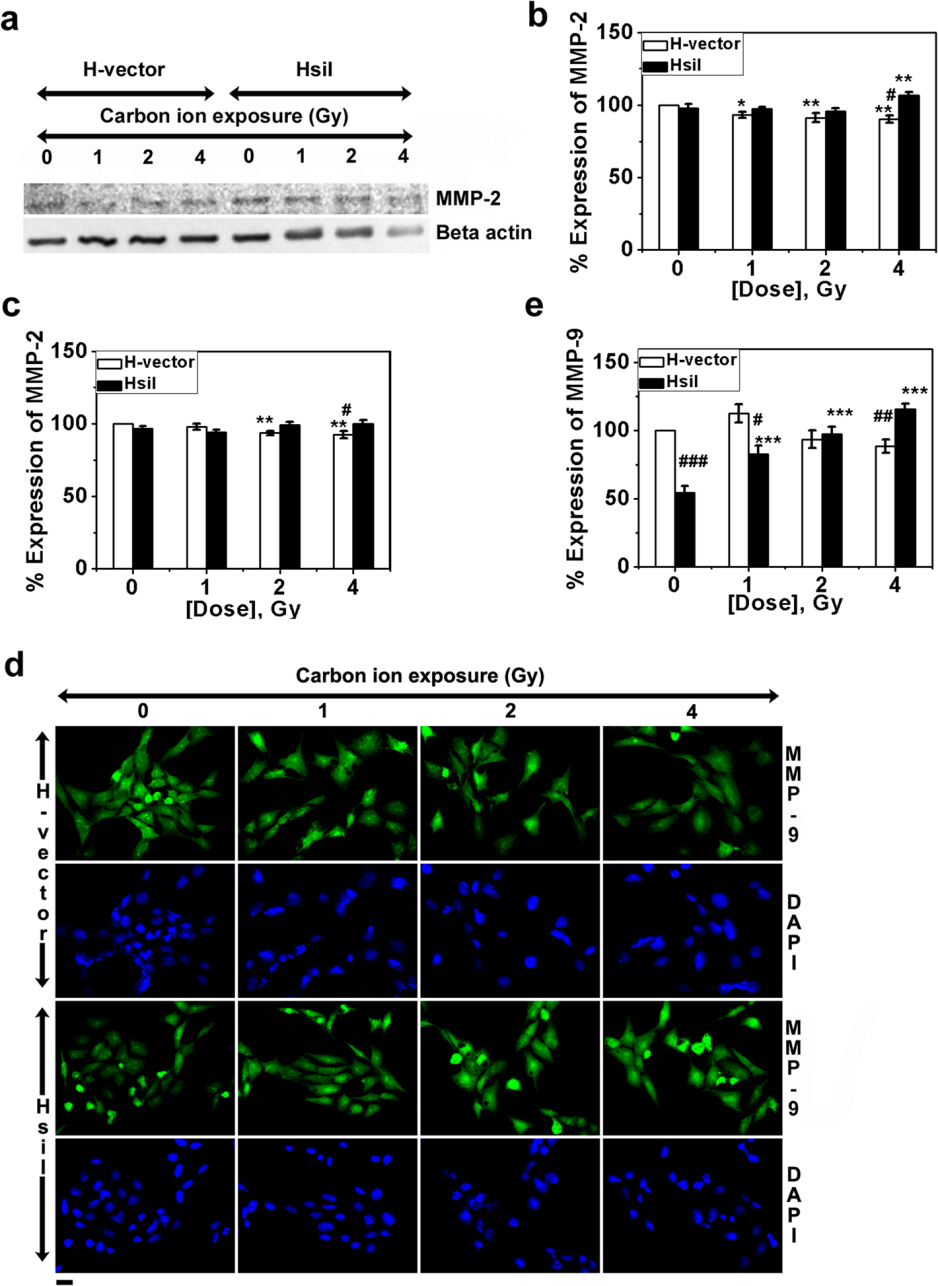
Expression of MMP-2 and MMP-9 protein after carbon ion exposure (0- 4 Gy) in H-vector and HsiI cells. **a** Typical western blot of MMP-2 and beta actin (used as internal control). **b** Band intensities of western blot of MMP-2 were measured by ImageJ software and normalized with respect to beta actin and plotted as % expression of MMP-2 considering MMP-2 expression in un-irradiated H-vector as 100 %. **c** Expression of MMP-2 was also measured by IF technique. Intensities of the immunostained cells for MMP-2 were measured by ImageJ software, normalized with respect to beta actin and plotted as mentioned above. Typical immunostained cells are shown in Additional file 2. **d** Expression of MMP-9 was measured by IF technique and typical immunostained cells are shown here. Scale bar represents 20 μm. **e** Intensities of the immunostained cells for MMP-9 were measured by ImageJ software, normalized with respect to beta actin and plotted as mentioned above. Each bar diagram represents the mean ± SD of three independent experiments

We have monitored activity of MMPs as given in earlier section. We also looked into the expression of these MMPs inside the cell by western blot and standard immunofluorescence (IF) technique. Beta actin was used as internal control. We observed a trend of slight decrease of MMP-2 expression in H-vector but practically there was no change in expression of MMP-2 in HsiI cells with or without carbon ion exposure. A western blot photograph of MMP-2 is shown in Fig. 3a. We have measured band intensities of MMP-2 of western blots and normalized with beta actin. Then normalized MMP-2 expression in un-irradiated H-vector was taken as 100 % and subsequently others were calculated and plotted in Fig. 3b. Almost similar pattern of expression of MMP-2 was observed by IF studies as shown in Fig. 3c and the IF images for MMP-2 expression were provided as Additional file 2. Notably, IF study showed knocking down of PARP-1 gene reduced expression of MMP-9 in un-irradiated HsiI cells and after irradiation it was increased dose-dependently as given in Fig. 3d and e, although the exact mechanism is not clear to us. As such there was no change in MMP-9 protein level in H-vector after carbon ion exposure.

### Expression of TIMP-1, TIMP-2 and TIMP-3 in cells after carbon ion exposure

**Fig. 4:**
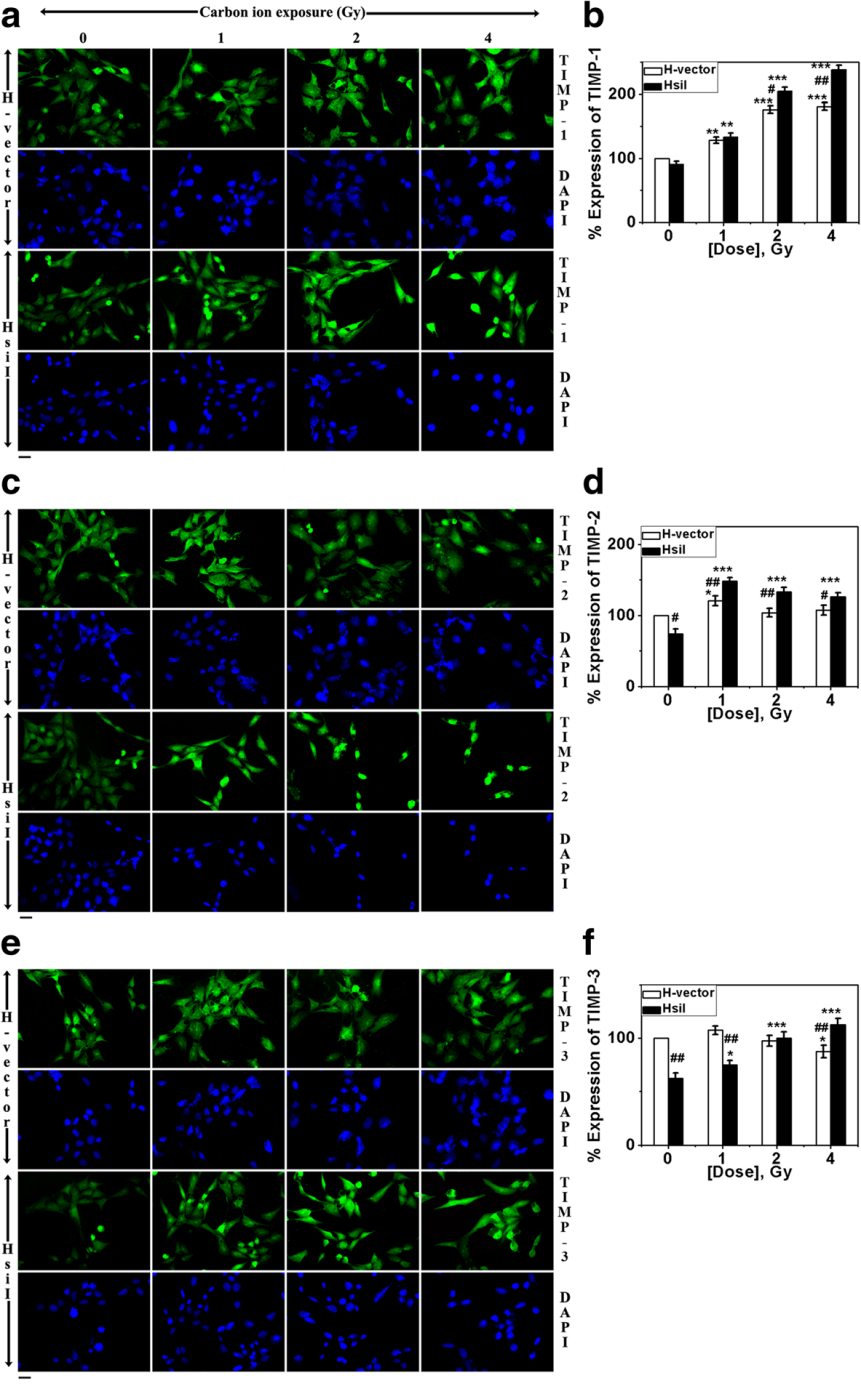
Studies of TIMP-1, TIMP-2 and TIMP-3 protein expression by IF after carbon ion exposure (0- 4 Gy). **a, c & e** Immunostained cells using anti-TIMP-1, anti-TIMP-2 and anti-TIMP-3 antibody followed by FITC conjugated host specific secondary antibody, are shown here respectively. DAPI was used to stain nucleus. Scale bar denotes 20 μm. **b, d & f** Intensities of the immunostained cells for TIMP-1, TIMP-2 and TIMP-3 respectively were measured by ImageJ software, normalized with beta actin. Normalized expression of respective protein in H-vector without irradiation was considered as 100 % expression of that protein. Accordingly rest were calculated and plotted. Each bar diagram represents the mean ± SD of three independent experiments. ‘**’ & ‘***’ denote 0.01 < *p* ≤ 0.05 & *p* ≤ 0.001 respectively for TIMPs expression at each of the particular dose compared with un-irradiated cell-types. ‘#’ & ‘##’ denote 0.01 < *p* ≤ 0.05 & 0.001 < *p* ≤ 0.01 respectively for difference of TIMPs expression between H-vector and HsiI at a particular dose

In general, MMPs facilitate metastasis in a particular cancer whereas TIMPs oppose it since TIMPs are endogenous inhibitor of MMPs by nature. We monitored the expression levels of TIMP-1, TIMP-2 and TIMP-3 by IF studies as shown in Fig. 4a, c and e. Here, beta actin was used as internal control to calculate the relative protein levels. The respective normalized protein level in un-irradiated H-vector was considered as 100 % expression and subsequently percentage of others samples were calculated and plotted. Three TIMPs showed different pattern of expression profile in both the cell type after carbon ion exposure. Only TIMP-1 expression increased dose-dependently whereas TIMP-2 and TIMP-3 remained unaltered after carbon ion exposure in H-vector as shown in Fig. 4b, d and f. On the contrary, TIMP-1 and TIMP-3 increased dose-dependently in HsiI cells after carbon ion exposure and their expression level was always significantly greater in HsiI cells compared with that in H-vector cells at higher doses (for TIMP-1 at and above 2 Gy whereas for TIMP-3 at 4 Gy). TIMP-2 expression level was significantly higher in HsiI cells compared with H-vector at all doses. These data implicated that overall TIMPs expression was higher in HsiI cells compared with H-vector after carbon ion exposure at higher doses. However, expression of TIMP-2 and TIMP-3 reduced significantly upon knocking down of PARP-1 gene without any irradiation and the reason is still unclear.

### Apoptosis is the predominant mode of cell death after carbon ion exposure

**Fig. 5:**
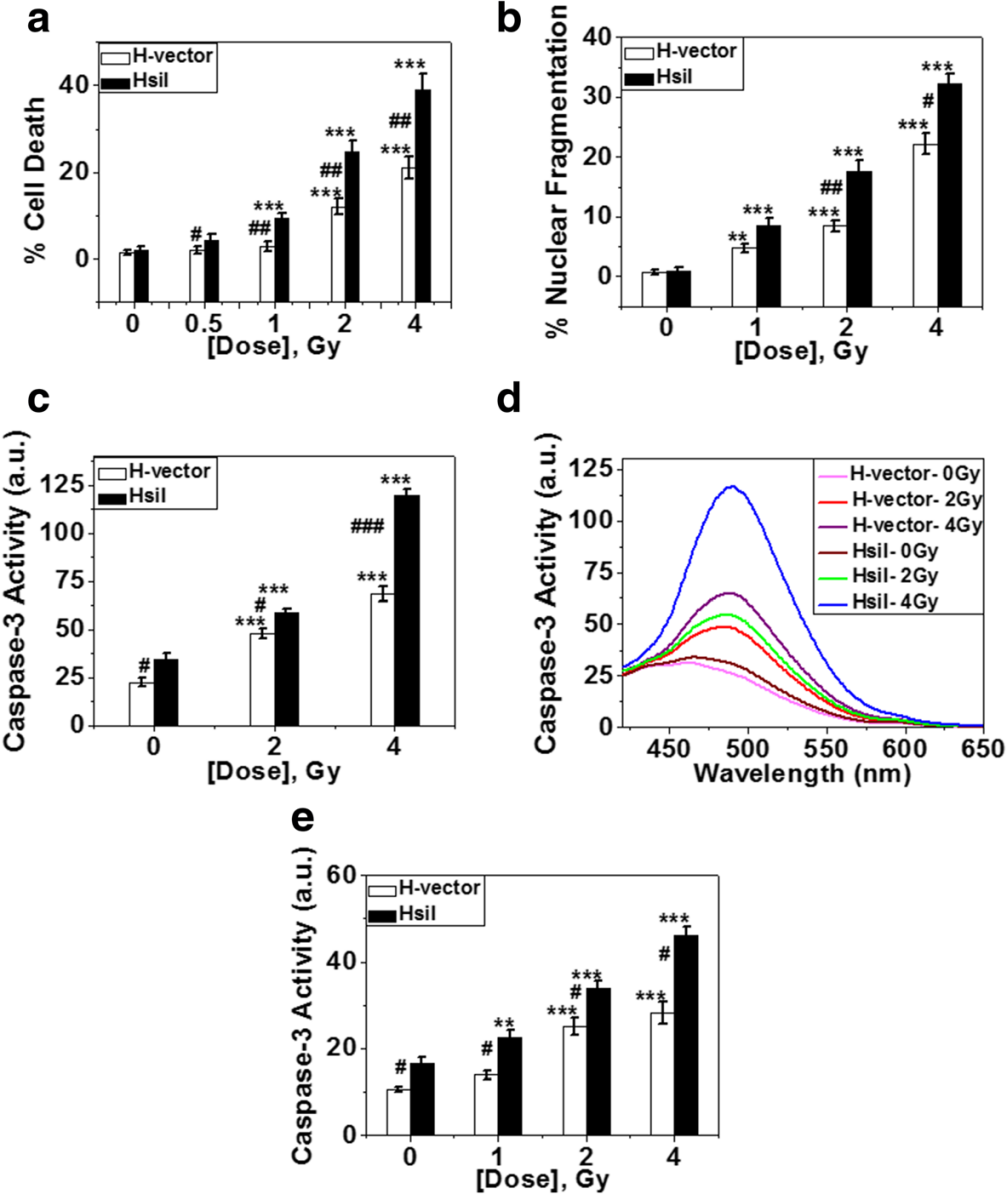
Study of different modes of cell death. **a** % of cell death as detected by trypan blue staining after 19 h of post-carbon ion exposure and plotted. Each bar diagram represents the mean ± SD of four independent experiments. **b** Detection of nuclear fragmentation. % nuclear fragmentation was calculated and plotted. Each bar diagram represents the mean ± SD of four independent experiments. Typical image of fragmented nucleus is given in Additional file 4a. **c & d** Assay of caspase-3 activity by fluorimetric study. Fluorimetric assay of caspase-3 activity of both H-vector and HsiI cells after 20 h of post-carbon ion exposure is shown here (**c**). Each bar diagram represents the mean ± SD of three independent experiments. The raw fluorescence spectra representing caspase-3 activity after carbon ion exposure is given here (**d**). **e** Caspase-3 activation by IF study. Fluorescence intensities of caspase-3 immunostained cells in 20 h post-carbon ion exposure were measured by ImageJ software and plotted. Each bar diagram represents the mean ± SD of three independent experiments. Typical photographs of immunostained cells are provided in Additional file 4b

Cell death after carbon ion exposure was measured by dye exclusion method using trypan blue. Dose-dependent increase of cell death was observed after carbon ion exposure in both cell types, but HsiI showed significantly higher sensitivity towards carbon ion exposure as shown in Fig. 5a.

We checked two modes of cell death - autophagy and apoptosis. Cleavage and lipidation of LC3B protein with phosphatidylethanolamine occur during autophagosome formation in cells undergoing autophagy and associates with the phagophores. This lapidated LC3 is well known as autophagy marker. In IF studies, it looks like vesicles in cytoplasm expressing LC3B or cells undergoing autophagy accumulate LC3 puncta. In our experimental condition, we didn’t find any autophagic cell death after carbon ion exposure in both H-vector and HsiI cells. No noticeable accumulation of LC3 puncta was observed in either cell-type with or without carbon ion exposure as shown in Additional file 3a. But chloroquine diphosphate, an artificial inducer of autophagy, treated H-vector cells showed the typical cytoplasmic vesicles formation or LC3 puncta in our experimental condition as shown in Additional file 3b. So, our experimental condition was able enough to detect any autophagic cell death.

Then we measured fraction of cells undergoing apoptotic death out of total cell death. At the late stage of apoptosis, nucleus of the cell becomes fragmented and condensed, forms characteristic apoptotic bodies. This is one of the hallmarks of apoptosis. Here, we measured nuclear fragmentation and it was increased dose-dependently in both the H-vector and HsiI cells after carbon ion exposure but it was always higher in HsiI cells than H-vector. Typical photographs of undamaged and fragmented nucleus are shown in Additional file 4a. These fragmented nuclei for each cell-type were counted and plotted to find % nuclear fragmentation as shown in Fig. 5b. At 4 Gy total cell death was about 23 % in H-vector and about 40 % in HsiI cells. Out of this total cell death apoptosis contributed about 23 % in H-vector cells and 33 % in HsiI cells. This data implicates that apoptosis is the predominant mode of cell death in each cell type under our experimental condition. We also confirmed apoptosis by measuring caspase-3 activation which was increased in a dose-dependent manner in both H-vector and HsiI cells after carbon ion exposure with respect to un-irradiated controls as shown in Fig. 5c. HsiI cells exhibited greater caspase-3 activity than H-vector suggesting HsiI cells showed more apoptosis induction. The raw spectra of fluorescence representing caspase-3 activity are also shown in Fig. 5d. We further checked caspase-3 activation by standard IF technique and observed the similar result as shown in Fig. 5e and Additional file 4b.

## Discussion

Conventional radiotherapy using gamma rays or X-rays is facing various difficulties like it lacks dose-localization to the target tumor cells, it grows radio-resistance, it is not effective for hypoxic tumors or deep seated tumors and importantly it triggers cancer metastasis by activating matrix metalloproteinases in various cancer cell-types as discussed in the Background section. On the contrary, high LET radiation or charged particles like carbon ion has better potential to suppress metastatic nature of cancer cells *in vitro* and *in vivo* [56, 57] including its other beneficial roles over low LET radiations as described earlier. But the detailed mechanism is not clear. Earlier we observed that PARP-1, a crucial DNA repair protein, plays regulatory roles in DNA damage responses after carbon ion exposure [43, 45]. Here, we have shown that PARP-1 is coming up as a key player in regulation of MMPs and TIMPs with or without carbon ion exposure which can be useful to deal the metastasis with a greater potential.

To check whether PARP-1 has any role in the regulation of metastatic nature of cancer cells after treatment with carbon ion exposure, we looked into the activities and expression of two major matrix metalloproteinases, MMP-2 and MMP-9, which are highly activated during cancer metastasis. We found high LET carbon ion exposure reduced both MMP-2 and MMP-9 activities dose-dependently in H-vector. These findings corroborated with other study where charged particles including carbon ion suppressed MMPs activities *in vitro* as well as *in vivo* [58]. There was a trend of decreased expression of MMP-2 and unaltered expression of MMP-9 after carbon ion exposure in H-vector cells. But the inhibition of PARP-1 by shRNA reduced the activity of MMP-2 and MMP-9 as well as the expression of MMP-9 protein without irradiation. Several lines of evidence supports our findings. PARP inhibitors like 5-AIQ and PJ-34 inhibit MMP-2 activity *in vitro* [47]. Inhibition of PARP with 5-AIQ down regulates the expression of MMP-9 and MMP-2 as well as their activities via decreasing NF-kB expression, facilitating the suppression of tumor metastasis in colorectal cancer [46]. But in our case, there is no significant change in MMP-2 protein level upon depletion of PARP-1 without any irradiation. Interestingly, inhibition of PARP-1 by PJ-34 or siRNA reduces the transcription of MMP-9 without changing MMP-2 expression [48]. To avoid non-specific interaction of PARP inhibitors, if any, we approached our studies using shRNA of PARP-1 and observed similar results as found from literature. Surprisingly, dose-dependent increase of MMP-9 protein level is noticed in HsiI cells although the enzymatic activity of the same is totally lost. We do not know the reason. Interestingly, Kauppinen and Swanson et al. showed PARP-1 activation facilitated MMP-9 release in the conditioned medium of microglial culture [59]. So it could be possible in our experimental condition that in spite of increased MMP-9 protein level, the activity of MMP-9 drastically reduced after carbon ion exposure due to the inhibition of its release into the conditioned medium in PARP-1 knocked down HsiI cells. However, the noteworthy observation is carbon ion exposure to these HsiI cells completely abolishes the activities of both MMP-2 and MMP-9 in almost all doses. This is the first report to show synergistic reduction of activities of MMPs by carbon ion exposure in combination with PARP-1 inhibition. Gamma irradiation significantly enhanced both MMP-2 and MMP-9 activities in H-vector cells supporting the previous reports as mentioned in Background section. But, PARP-1 depletion did not show any significant enhancement of MMPs activities after gamma irradiation in our experimental condition, implicating the beneficial role of PARP-1 inhibition in gamma therapy. So, our data implicates that better anti-metastatic effect can be obtained for cervical cancer cells when PARP-1 inhibition is combined with carbon ion exposure.

There are various other factors related to the metastatic potential of cancer cells. Out of those, the TIMPs – the endogenous inhibitors of MMPs, are also very crucial candidates to control metastasis. Here, we measured TIMP-1, TIMP-2 and TIMP-3 protein expression by IF technique in both H-vector and HsiI cell types with or without carbon ion exposure. Previous reports show the up-regulation of TIMP-1 in different cell types using low LET ionizing radiations as well as particle radiations [27, 34]. TIMP-1 over-expression radio-sensitizes the renal cancer cells to gamma radiation and also shows elevation of TIMP-2 protein level as well as decrease of MMP-2 activity [29]. On the contrary, there are other reports opposing these observations. One investigation demonstrates no significant change in TIMP-1 and TIMP-2 in Panc-1, pancreatic cancer cells after gamma irradiation [28]. Again, low LET ionizing radiation has no effect on TIMP-2 expression in human lung epithelial cells [24]. Since the low LET radiation and high LET carbon ion exposure are totally different in terms of their physical properties, they show different biological effects. Very little is known about cellular responses against high LET carbon ion exposure. Our results show that up-regulation of TIMP-1 after carbon ion exposure although expression of TIMP-2 and 3 remains the same in H-vector cells. But, dose-dependent increase of TIMP-1 and TIMP-3 is observed in HsiI cells and their expressions are significantly higher in HsiI cells than that in H-vector at higher dose such as at 4 Gy. Furthermore, TIMP-2 expression is significantly higher in HsiI than H-vector at all doses. It is to be noted that TIMP-1 and TIMP-2 are the endogenous inhibitors of MMP-9 and MMP-2 respectively. So their up-regulation might be explained with the reduction of respective MMPs activities, although we didn’t check the activities of TIMPs in our experimental conditions. This data further implicates that overall anti-metastatic effect as detected by higher expression of TIMPs obtained by combination of PARP-1 inhibition with carbon ion exposure is far better than that by carbon ion exposure alone. In other words, PARP-1 inhibition potentiates anti-metastatic effect of carbon ion exposure in terms of reduced activities of MMPs and higher expression of TIMPs. Our work further raises few interesting questions which remained unexplored. The expression patterns of all three TIMPs are different after carbon ion exposure with or without PARP-1 inhibition. We do not know why TIMP-2 and TIMP-3 are down-regulated in PARP-1 knocked down cells without any radiation. TIMP-1 remains unchanged after knocking down of PARP-1 gene in our experimental condition. However, decreased transcription of TIMP-1 is noticed after PARP-1 inhibition by inhibitor or siRNA in cardiac fibroblasts [48]. It is found that deletion or knockout of PARP-1 gene enhances TIMP-2 expression as detected by RT-PCR [60, 61], although they do not mention the protein level of TIMP-1 and TIMP-2. PARP-1 gene deletion can increase the expression of TIMP-3 to a lower extent whereas status of TIMP-3 in protein level is not reported [61]. We observe carbon ion exposure up-regulates TIMP-3 level dose-dependently in HsiI cells but not H-vector and expression of TIMP-3 is significantly higher in HsiI cells than H-vector at 4 Gy. TIMP-3 is reported to increase apoptosis upon over-expression [62]. In another study, overexpression of TIMP-3 in HeLa carcinoma cell line induces a FADD-dependent type II apoptotic signaling pathway [63]. Hence, increased level of TIMP-3 in HsiI cells can be associated with higher apoptosis induction after carbon ion exposure. Moreover, we showed knocking down of PARP-1 can also activate extrinsic pathway of apoptosis more than the wild type after carbon ion exposure in our previous study [45].

Furthermore, we investigated the mode of cell death taken by the cells when the metastatic potential was compromised upon knocking down of PARP-1 with combination of carbon ion exposure. We mainly checked apoptosis and autophagy induction. HsiI cells having lower MMPs activities showed higher amount of apoptosis induction in terms of caspase-3 activation and nuclear fragmentation than H-vector after irradiation with carbon ion exposure, consistent with our previous reports [43, 45]. Notably, we observe that % cell death, as detected by trypan blue is comparable or slightly higher than % nuclear fragmentation after irradiation with carbon ion exposure. This data implicates that most of the cells dies through apoptosis pathway after carbon ion exposure. Surprisingly, we do not see any significant amount of autophagy after carbon ion exposure in both cell types in our experimental condition. But there are reports where carbon ion exposure induced autophagy [64, 65] and PARP-1 is also involved in autophagic cell death [66, 67]. Our data shows apoptosis is the major mode of cell death after carbon ion exposure in combination with PARP-1 depletion.

## Conclusions

Our results suggest that PARP-1 depletion along with carbon ion exposure synergistically increases apoptosis and decreases metastatic properties via reducing activities of both MMP-2 and MMP-9 and overall up-regulation of TIMP-1, TIMP-2 and TIMP-3 in HeLa cells. PARP-1 inhibition has beneficial role via stopping increase of metastatic property induced by gamma irradiation. So, PARP-1 inhibition in combination with carbon ion exposure could be a promising tool in hadrontherapy for treatment of cervical or other cancers and could be beneficial in post-irradiation stages by stopping metastasis.

## Additional files

Additional file 1: Loading control of gelatin zymography. a A representative gel image of SDS-PAGE without gelatin run parallel to the gelatin zymography gel with same amount of samples under same experimental condition as given in Fig. 2a after carbon ion exposure (0- 4 Gy) is shown here. This was treated as loading control for normalization of the band intensities from the zymography gel to get the MMPs activities. ‘M’ denotes the molecular weight marker lane. b A representative gel image of loading control for gamma irradiated samples as given in Fig. 2d. (TIF 220 kb) Additional file 2: MMP-2 expression after carbon ion exposure (0- 4 Gy) by IF. Typical photographs of immunostained cells of both H-vector and HsiI are shown here. Mouse anti-MMP-2 primary antibody was used, followed by the secondary antibody of FITC tagged goat anti-mouse IgG_1_ to detect the MMP-2 expression. DAPI was used to stain the nucleus. Scale bar represents 20 μm. (TIF 484 kb) Additional file 3: Detection of autophagy by IF. a Typical photograph of immunostained H-vector and HsiI cells with LC3B polyclonal antibody detected by donkey anti-rabbit IgG-R (tagged with Rhodamine) after carbon ion exposure at 4 Gy is given here and DAPI is used to stain nucleus. b Typical photograph of H-vector cells undergoing autophagy after treatment with 50 μM chloroquine diphosphate for 16 h at 37 °C followed by immunostaining with LC3B polyclonal antibody is shown here as positive control. Scale bar represents 20 μm. One representative cell is zoomed to show the LC3 puncta. (TIF 718 kb) Additional file 4: Apoptosis induction after carbon ion exposure (0- 4 Gy). a Detection of nuclear fragmentation. Typical photographs of undamaged nuclei and fragmented nuclei of H-vector and HsiI cells irradiated with 4 Gy of carbon ion exposure after staining with Hoechst dye are shown here. One representative cell is zoomed to show the fragmented nucleus or apoptotic bodies. b Typical photographs of immunostained cells of caspase-3 (Casp-3) activation in both H-vector and HsiI as detected by standard IF technique are shown here. Rabbit anti-Casp-3 antibody was detected by the secondary antibody of Rhodamine tagged (Rhd) donkey anti-rabbit IgG. DAPI was used to counterstain the nucleus. Scale bar represents 20 μm. (TIF 947 kb)

## Abbreviations

DAPI: 4’, 6-diamidino-2-phenylindole
FITC: Fluorescein isothiocyanate
IF: Immunofluorescence
LET: Linear energy transfer
MMPs: Matrix metalloproteinases
PARP-1: Poly(ADP-ribose) polymerase-1
SD: Standard deviation
TIMPs: Tissue inhibitor of matrix metalloproteinases
