# Supplementary figures and images for: PARP-1 depletion in combination with carbon ion exposure significantly reduces MMPs activity and overall increases TIMPs expression in cultured HeLa cells
Source: Radiat Oncol. 2016 Sep 22;11:126. doi: 10.1186/s13014-016-0703-x (PMC5034624; doi:10.1186/s13014-016-0703-x)

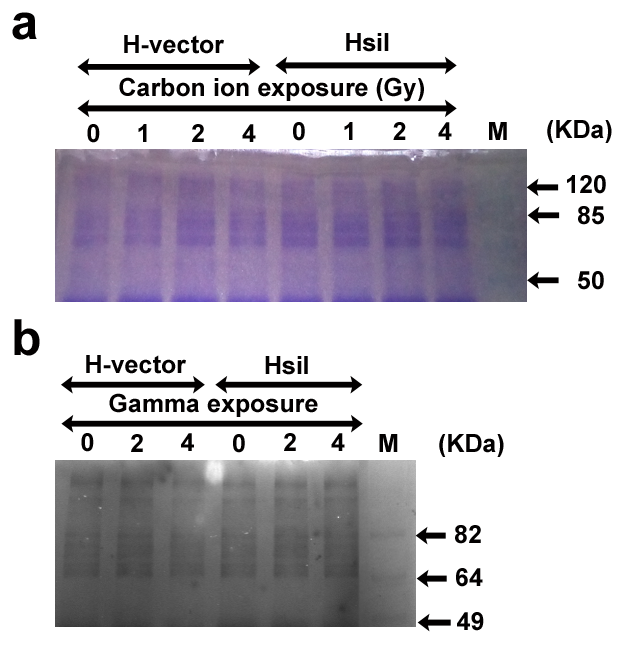

Supplement: Additional file 1: — Loading control of gelatin zymography. a A representative gel image of SDS-PAGE without gelatin run parallel to the gelatin zymography gel with same amount of samples under same experimental condition as given in Fig. 2a after carbon ion exposure (0- 4 Gy) is shown here. This was treated as loading control for normalization of the band intensities from the zymography gel to get the MMPs activities. ‘M’ denotes the molecular weight marker lane. b A representative gel image of loading control for gamma irradiated samples as given in Fig. 2d. (TIF 220 kb) [file 13014_2016_703_MOESM1_ESM.tif]

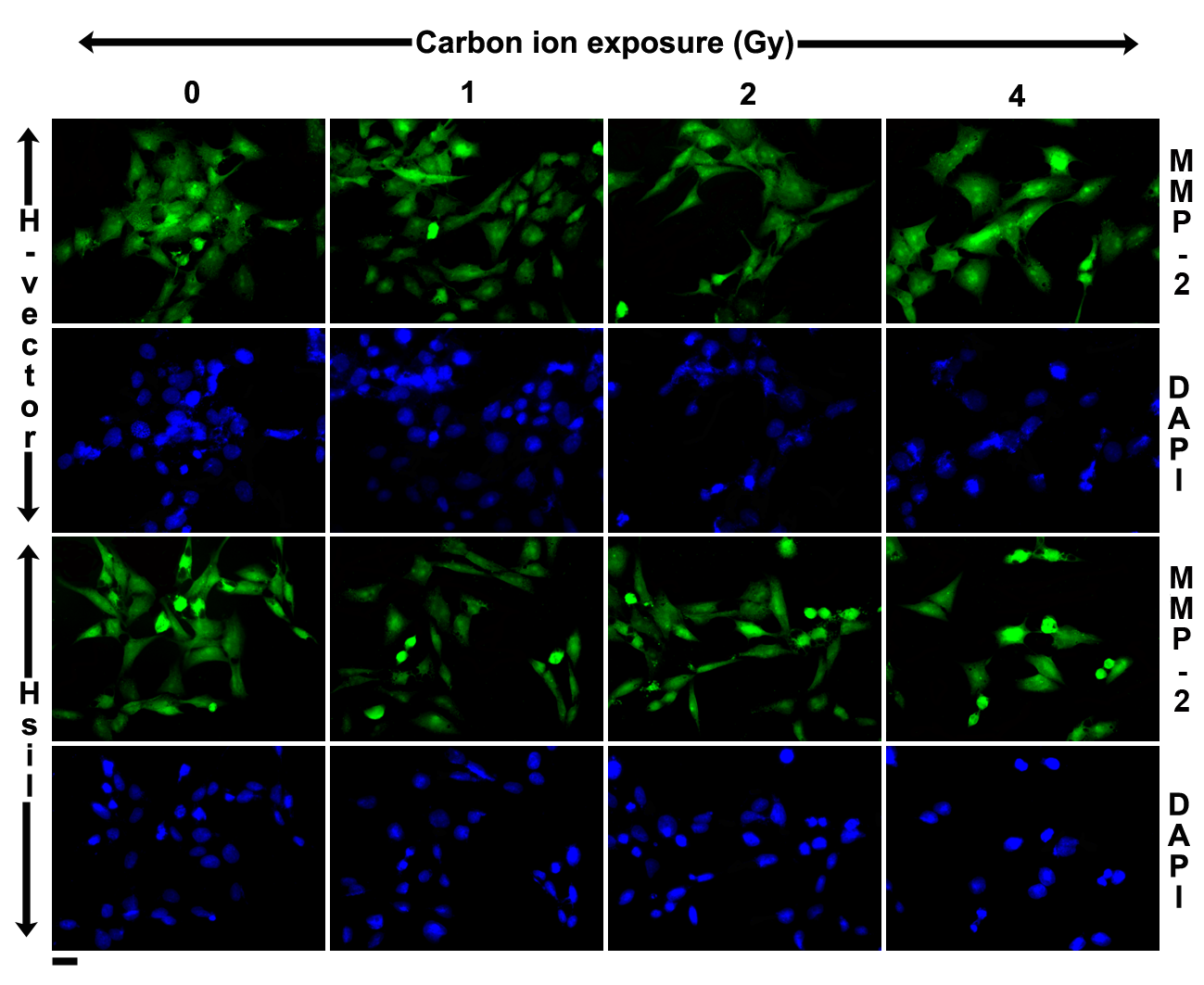

Supplement: Additional file 2: — MMP-2 expression after carbon ion exposure (0- 4 Gy) by IF. Typical photographs of immunostained cells of both H-vector and HsiI are shown here. Mouse anti-MMP-2 primary antibody was used, followed by the secondary antibody of FITC tagged goat anti-mouse IgG1 to detect the MMP-2 expression. DAPI was used to stain the nucleus. Scale bar represents 20 μm. (TIF 484 kb) [file 13014_2016_703_MOESM2_ESM.tif]

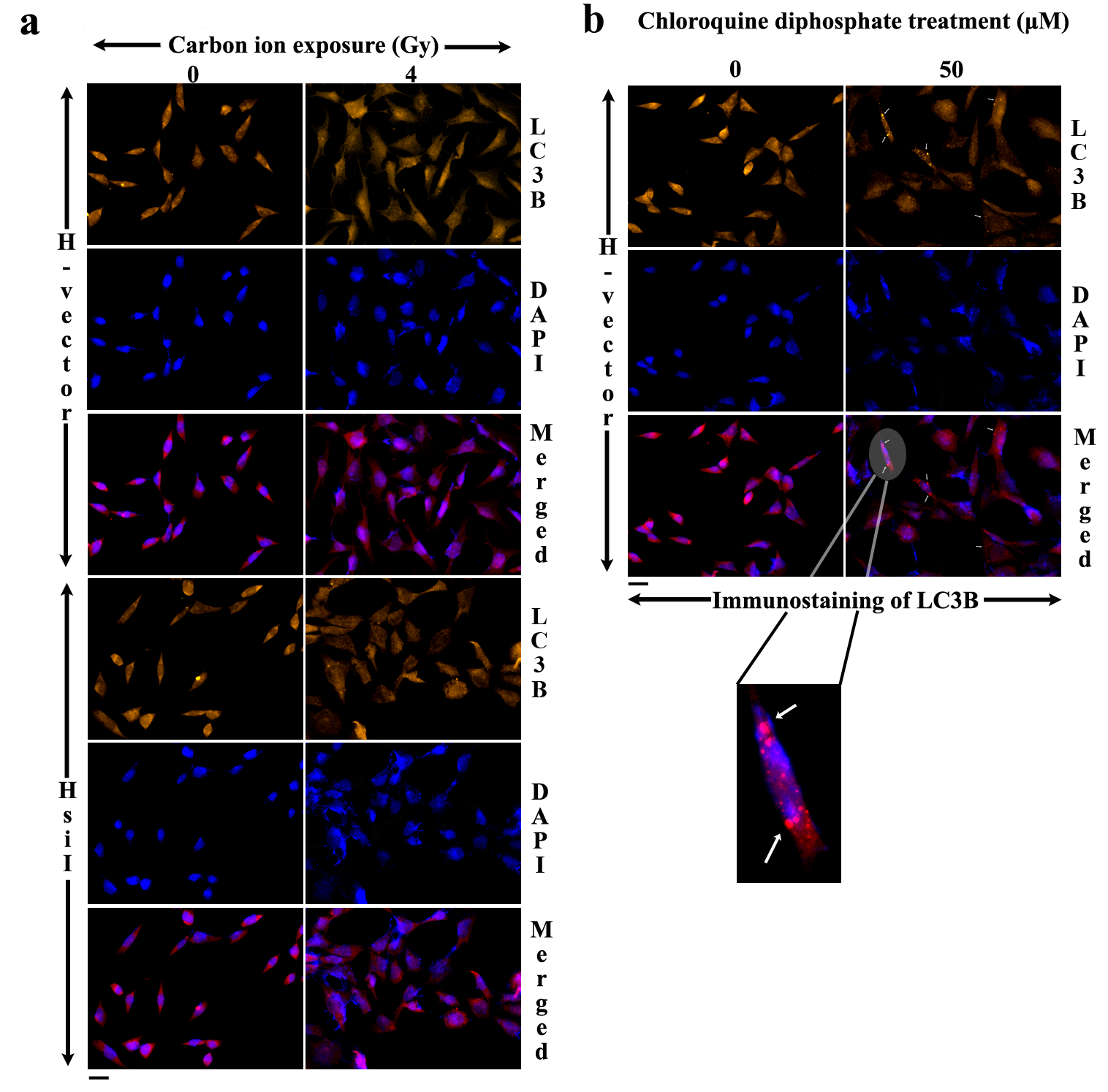

Supplement: Additional file 3: — Detection of autophagy by IF. a Typical photograph of immunostained H-vector and HsiI cells with LC3B polyclonal antibody detected by donkey anti-rabbit IgG-R (tagged with Rhodamine) after carbon ion exposure at 4 Gy is given here and DAPI is used to stain nucleus. b Typical photograph of H-vector cells undergoing autophagy after treatment with 50 μM chloroquine diphosphate for 16 h at 37 °C followed by immunostaining with LC3B polyclonal antibody is shown here as positive control. Scale bar represents 20 μm. One representative cell is zoomed to show the LC3 puncta. (TIF 718 kb) [file 13014_2016_703_MOESM3_ESM.tif]

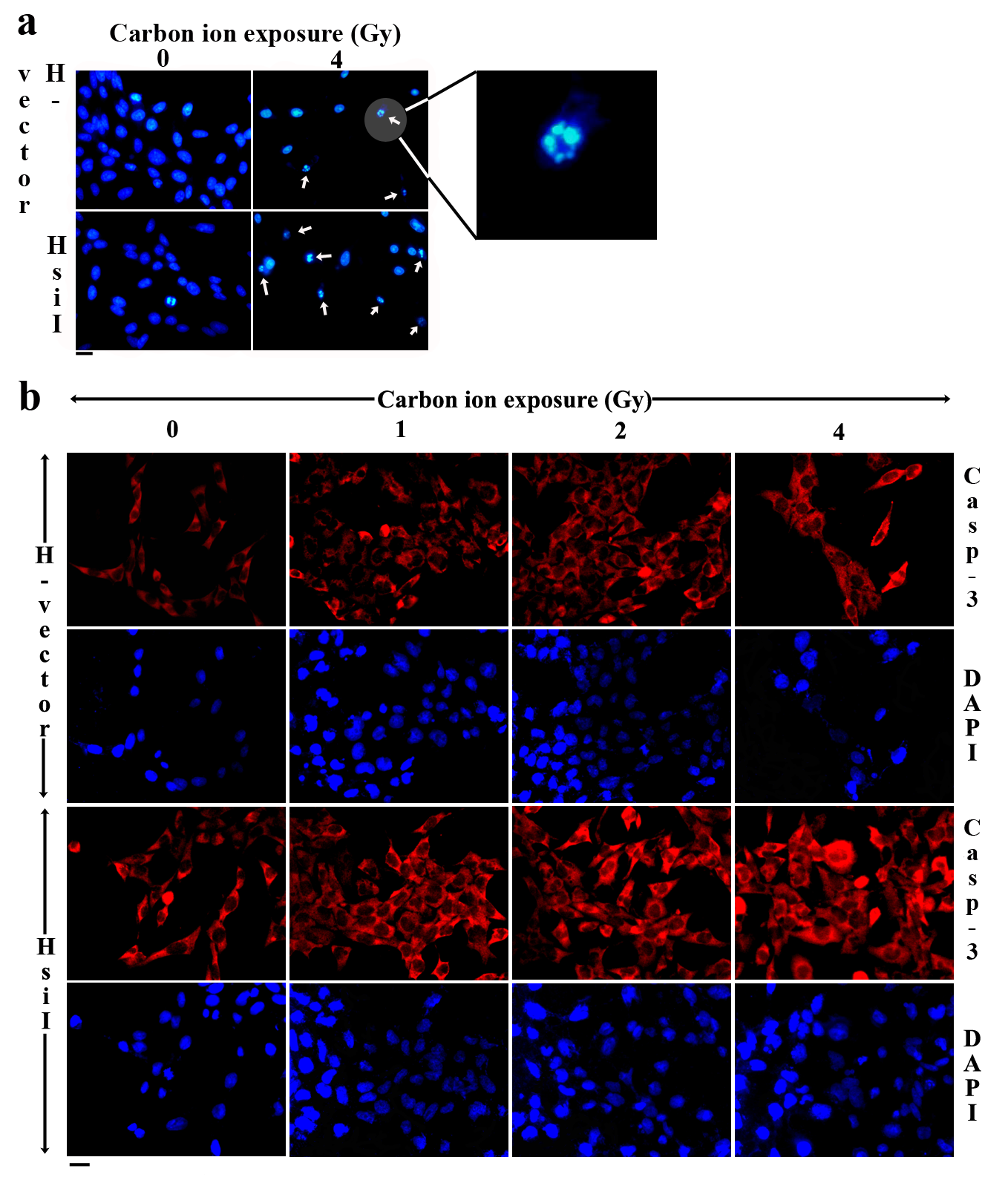

Supplement: Additional file 4: — Apoptosis induction after carbon ion exposure (0- 4 Gy). a Detection of nuclear fragmentation. Typical photographs of undamaged nuclei and fragmented nuclei of H-vector and HsiI cells irradiated with 4 Gy of carbon ion exposure after staining with Hoechst dye are shown here. One representative cell is zoomed to show the fragmented nucleus or apoptotic bodies. b Typical photographs of immunostained cells of caspase-3 (Casp-3) activation in both H-vector and HsiI as detected by standard IF technique are shown here. Rabbit anti-Casp-3 antibody was detected by the secondary antibody of Rhodamine tagged (Rhd) donkey anti-rabbit IgG. DAPI was used to counterstain the nucleus. Scale bar represents 20 μm. (TIF 947 kb) [file 13014_2016_703_MOESM4_ESM.tif]
